# Trends in Liver Transplantation for Acute Alcohol-Associated Hepatitis During the COVID-19 Pandemic in the US

**DOI:** 10.1001/jamanetworkopen.2021.18713

**Published:** 2021-07-29

**Authors:** Therese Bittermann, Nadim Mahmud, Peter Abt

**Affiliations:** 1 Perelman School of Medicine, University of Pennsylvania, Philadelphia

## Abstract

This cohort study examines rates of adding patients with acute alcohol-associated hepatitis to the liver transplant waiting list and receipt of transplants by these patients in the US during the COVID-19 pandemic compared with before the pandemic.

## Introduction

Alcohol consumption and alcohol-related hospitalizations in the US have increased since the start of the COVID-19 pandemic.^1,2^ The broader public health consequences associated with these trends are incompletely characterized. Liver transplantation has become an accepted treatment for severe, acute alcohol-associated hepatitis.^3^ We describe trends in liver transplantation for acute alcohol-associated hepatitis before and during the COVID-19 pandemic.

## Methods

This cohort study was deemed exempt by the University of Pennsylvania Institutional Review Board with a waiver of informed consent under Common Rule Exemption 4. This study adheres to the Strengthening the Reporting of Observational Studies in Epidemiology (STROBE) reporting guideline.

This retrospective cohort included adults on the national liver transplant waiting list (with or without simultaneous kidney transplant) between March 1, 2018, and February 28, 2021. The cohort was developed using the United Network for Organ Sharing (UNOS) database. Candidates listed with acute alcohol-associated hepatitis were identified using UNOS diagnostic coding. Rates of new listings and completed liver transplants for patients with acute alcohol-associated hepatitis before the COVID-19 pandemic (ie, March 1, 2018, to February 29, 2020) vs during the pandemic (ie, March 1, 2020, to February 28, 2021) were compared, as were the sociodemographic characteristics of patients with acute alcohol-associated hepatitis, given suspected disparities in access to mental health care.^4^ Race/ethnicity was self-reported by patients. Descriptive comparisons used χ^2^ and Kruskal-Wallis tests. Using linear regression, smoothed estimates of the percentage change in patients with acute alcohol-associated hepatitis added to the liver transplant waiting list and receiving transplants over time were plotted relative to the prepandemic model’s intercept as the baseline. A difference-in-differences approach estimated the pandemic’s association with monthly changes in patients with acute alcohol-associated hepatitis being added to the waiting list and receiving liver transplants, accounting for secular trends. The a priori hypothesis was that changes in waiting list additions for acute alcohol-associated hepatitis would begin at the start of the COVID-19 pandemic (ie, March 2020). However, visual inspection of month-by-month trends indicated that deviations began June 2020. Therefore, the difference-in-differences analysis compared the number of patients with acute alcohol-associated hepatitis added to the waiting list and receiving liver transplantation between June 1, 2020, and February 28, 2021 (exposed group), vs June 1, 2020, to February 29, 2020 (unexposed group), relative to each group’s control period (ie, March 1, 2020, to May 30, 2020, and March 1, 2019, to May 30, 2019, respectively). *P* values were 2-sided, and statistical significance was set at *P* < .05. Data were analyzed in March 2021.

## Results

Between March 1, 2018, and February 28, 2021, 606 candidates (median [interquartile range] age, 41 [35-49] years; 401 [66.1%] men) with acute alcohol-associated hepatitis were identified among 38 217 patients on the liver transplant waiting list. From March 2020 to February 2021, the mean rate of adding patients with acute alcohol-associated hepatitis to the liver transplant waiting list was 2.3% (0.7%), and their rate of receipt of liver transplantation was 4.4% (1.9%) (Figure, A). This represented a mean increase by 106.6% in additions to the waiting list (*P* < .001) and 210.2% in receipt of liver transplant (*P* < .001) compared with March 2018 through February 2020 (Figure, B). At maximum follow-up (ie, February 2021), the relative increase was 105.6% in waiting list additions and 411.8% in receipt of liver transplant. There was no difference between patients with acute alcohol-associated hepatitis on the liver transplant waiting list in the prepandemic vs during pandemic groups in sex (216 men [63.3%] vs 185 men [70.1%]; *P* = .08), age (median [interquartile range] age, 41 [35-49] years vs 40 [35-49] years; *P* = .81), race/ethnicity (272 White patients [79.8%] vs 212 White patients [80.3%]; 18 Black patients [5.3%] vs 10 Black patients [3.8%]; 30 Hispanic patients [8.8%] vs 24 Hispanic patients [9.1%]; 21 patients with other race/ethnicity [including Asian, Pacific Islander, Alaska Native, and multiracial] [6.2%] vs 18 patients with other race/ethnicity [6.8%]; *P* = .84), or insurance type (private insurance: 213 patients [62.5%] vs 160 patients [60.6%]; Medicaid: 100 patients [29.3%] vs 86 patients [32.6%]; Medicare and other: 28 patients [8.2%] vs 18 patients [6.8%]; *P* = .61).

**Figure.**
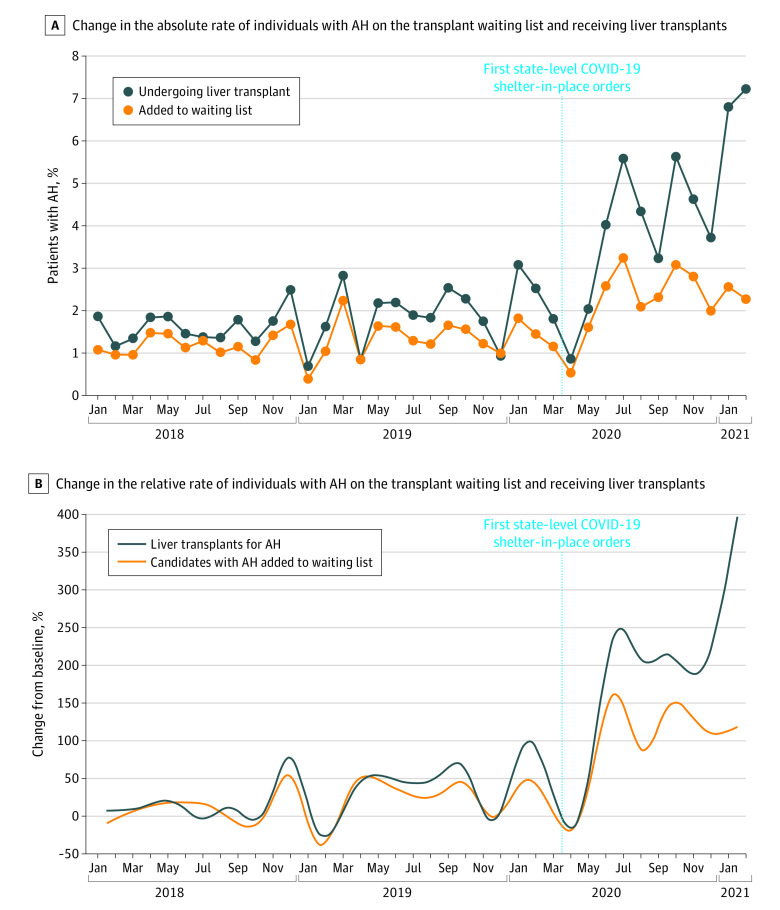
Change in the Rates of Additions to the Liver Transplant Waiting List and Receipt of Liver Transplants Among Patients With Acute Alcohol-Associated Hepatitis (AH) Nationally From March 2018 through February 2021

In difference-in-differences analyses, the rate of adding patients with acute alcohol-associated hepatitis to the liver transplant waiting list was a mean (SE) of 18.00 (4.37) listings per month higher from June 2020 to February 2021 compared with the expected counterfactual trend (*P* = .001) (Table). Liver transplants for acute alcohol-associated hepatitis were a mean (SE) of 13.11 (4.32) transplants per month higher during this period (*P* = .007). These figures represent a 325.0% increase in patients added to the waiting list and 268.5% increase in patients receiving a liver transplant compared with the expected counterfactual.

**Table.** Analysis of Monthly Additions to the Liver Transplant Waiting List and Receipt of Transplants Among Patients With Alcohol-Associated Hepatitis

	AH additions to the waiting list per mo, mean (SE)^a^		Difference		AH LTs per mo, mean (SE)^a^		Difference	
	During control period (March-May)	During change period (June-February)	Mean (SE)	*P* value	During control period (March-May)	During change period (June-February)	Mean (SE)	*P* value
March 2019-February 2020	16.67 (2.67)	14.67 (1.54)	−2.00 (3.09)	.52	12.00 (2.65)	12.11 (1.53)	0.11 (3.06)	.97
March 2020-February 2021	10.00 (2.67)	26.00 (1.54)	16.00 (3.09)	<.001	7.67 (2.65)	20.89 (1.53)	13.22 (3.06)	<.001
Difference-in-differences^b^	NA	NA	18.00 (4.37)	.001	NA	NA	13.11 (4.32)	.007

Abbreviations: AH, acute alcohol-associated hepatitis; LT, liver transplantation.

^a^ Visual inspection of changes in baseline AH listings and transplants during the pandemic demonstrated an increase beginning June 2020. Thus, the control period was designated as March through May. The change period was designated as June through February of the following year.

## Discussion

This cohort study found that the number of patients awaiting and undergoing liver transplantation for acute alcohol-associated hepatitis substantially increased during the COVID-19 pandemic. This marked change is likely associated with recent patterns in high-risk alcohol use.^1,2^ Additional contributions may have included transplant centers’ increasing acceptance of pursuing liver transplantation for acute alcohol-associated hepatitis^3^ and a revision of the liver transplantation allocation system in February 2020,^5^ as well as possibly concurrent lifestyle changes and COVID-19 infection itself. Nevertheless, these recent recipients of liver transplant will require intensive longitudinal multidisciplinary care to reduce their risk of alcohol relapse and ensure successful outcomes.

Study limitations include the potential underestimation of patients with acute alcohol-associated hepatitis awaiting and undergoing liver transplantation.^6^ Geographic differences could not be evaluated owing to sample size constraints.

Liver transplantation selection practices during the COVID-19 pandemic have reflected the worrisome trends in alcohol consumption observed nationally. The COVID-19 pandemic’s impact on the future role of liver transplantation for both acute and chronic alcohol-related liver disease should be explored.
